# Effects of Acrylamide on Mouse Implantation and Decidualization

**DOI:** 10.3390/ijms26094129

**Published:** 2025-04-26

**Authors:** Hong-Yuan Yang, Hui-Na Luo, Zai-Mei Wang, Dan-Dan Jin, Zeng-Ming Yang

**Affiliations:** 1 Key Laboratory of Animal Genetics, Breeding and Reproduction in the Plateau Mountain Region, College of Animal Science, Guizhou University, Guiyang 550025, China; 2 College of Veterinary Medicine, South China Agricultural University, Guangzhou 510642, China

**Keywords:** acrylamide, implantation, decidualization, oxidative stress, ferroptosis

## Abstract

Acrylamide is a class 2A carcinogen with neurotoxicity and genotoxicity. In addition to industrial production, it is ubiquitous in high-temperature heated high-carbohydrate foods. Numerous studies have confirmed the toxicity of ACR on reproduction. Implantation and decidualization are crucial processes during the establishment of pregnancy in rodents and humans. However, its effect on uterine implantation and decidualization remains poorly understood. The objective of this study is to elucidate the mechanism by which ACR affects implantation and decidualization in mice. ACR is exposed in the daily drinking water of female mice, and the dose is calculated according to the body weight of the mice. After 3 months of administration at concentrations of 0, 20, and 30 mg ACR/kg/d, female mice are mated with male mice to induce pregnancy. Compared to the control group, ACR treatment significantly reduces the number of embryo implantations and litter size. ACR treatment leads to abnormal expression of endometrial receptivity-related molecules in the luminal epithelium on day 4 of pregnancy, including a decrease in p-STAT3 level and an increase in MUC1 and MSX1 levels. The level of decidualization-related molecules is obviously downregulated by ACR. Furthermore, ACR treatment results in abnormality of oxidative stress- and ferroptosis-related protein levels at the implantation site on day 5. In conclusion, acrylamide can impair mouse implantation and decidualization by disrupting oxidative stress and ferroptosis.

## 1. Introduction

Embryo implantation and decidualization are critical for pregnancy establishment and maintenance in rodents and humans [1]. Abnormalities during implantation and decidualization can impede placental development and result in pregnancy complications such as recurrent miscarriage, preeclampsia, and intrauterine growth restriction [2].

Acrylamide (ACR) is a water-soluble vinyl monomer with a variety of chemical and industrial applications [3]. In addition to artificial synthesis, ACR is primarily formed through the reaction of amino acids and reducing sugars (at temperatures exceeding 120 °C) under conditions of high temperature and heat in food processing. Foods such as potatoes, bread, and coffee undergo heating or baking processes, resulting in the formation of ACR [3,4]. The amount of ACR in these foods can reach up to 2300 μg/kg [5]. ACR is considered to be a neurotoxic, genotoxic, and carcinogenic substance with potential reproductive toxicity [6]. ACR impedes the development of mouse oocytes, induces abnormal meiosis, enhances apoptosis of cumulus cells, and causes a 2-cell block in mouse embryos [7,8,9,10]. ACR can also induce ovarian dysfunction and inhibit placental development [11,12]. Although a previous study showed that ACR can inhibit mouse endometrial decidualization [13], the precise mechanism by which ACR influences implantation and decidualization remains poorly elucidated.

During pregnancy, the production of reactive oxygen species (ROS) is elevated due to increased metabolic activity in placental mitochondria and fetal metabolic demands during growth, thus constituting an inherent state of oxidative stress [14]. Physiological levels of oxidative stress play a crucial role in the reproductive process. Reactive oxygen species regulate implantation, embryogenesis, and pregnancy [15,16]. When the equilibrium between oxidants and antioxidants is disrupted, abnormal intracellular ROS levels are related to stillbirth, recurrent spontaneous abortion, preeclampsia, fetal growth restriction, preterm delivery, and fetal malformations [17,18].

Excessive ROS can induce oxidative stress, which further triggers ferroptosis [19]. Ferroptosis is a distinct form of iron-dependent non-apoptotic cell death, playing a crucial role during pregnancy, influencing the processes of embryogenesis, implantation, and pregnancy maintenance [20]. ACR can induce oxidative stress and cause damage to nerve cells [21,22,23]. ACR-treated rats exhibit a significant decrease in renal antioxidant markers SOD and GSH [24]. ACR can induce oxidative stress-driven autophagy to trigger ferroptosis, consequently leading to liver injury [25]. Furthermore, ACR treatment induces ferroptosis in HSC-T6 cells [26]. Even if ACR is implicated in oxidative and ferroptosis, it is unknown whether ACR affects oxidative stress and ferroptosis during early pregnancy.

In this study, we examined the effects of ACR on implantation and decidualization after female mice were exposed to ACR for 3 months. The results indicate that ACR treatment leads to aberrant expression of implantation and decidualization marker molecules, oxidative stress- and ferroptosis-related molecules. These alterations are associated with adverse effects on early pregnancy and pregnancy outcomes.

## 2. Results

### 2.1. The Deleterious Effects of Acrylamide on Implantation and Pregnancy Outcomes

**Figure 1:**
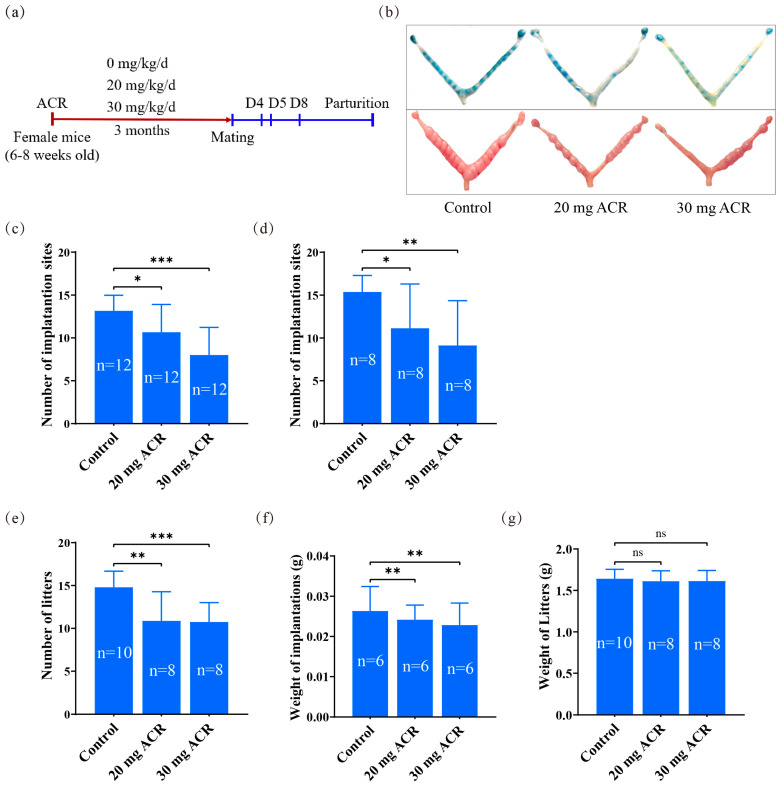
Effects of ACR on mouse pregnancy. (**a**) Diagram of ACR treatment protocol. (**b**) The morphology of mouse uteri on days 5 and 8 of pregnancy treated with 0, 20 mg ACR/kg/d (20 mg ACR), and 30 mg ACR/kg/d (30 mg ACR) for 3 months. (**c**) The number of implantation sites on day 5 of pregnancy in each group. (**d**) The number of implantation sites on day 8 of pregnancy in each group. (**e**) The number of newborn mice in each group. (**f**) The weight of implantation sites on day 8 in each group. (**g**) The weight of newborn mice in each group. * *p* <  0.05; ** *p* <  0.01; *** *p* <  0.001; ns: no significant.

Following 3 months of ACR exposure in drinking water, the female mice were mated with male mice to induce pregnancy (Figure 1a). In comparison with the control group, the number of implantation sites in ACR-treated groups (20 mg/kg/d and 30 mg/kg/d) was significantly reduced on days 5 and 8, and the litter size was also significantly reduced (Figure 1b–e). Additionally, the weight of implantation sites on day 8 was significantly reduced in the ACR-treated groups; however, no significant changes in the body weight of newborn mice were observed (Figure 1f,g). These results indicated that acrylamide exposure was detrimental to pregnancy in mice.

### 2.2. The Effects of Acrylamide on Ovarian Hormones

**Figure 2:**
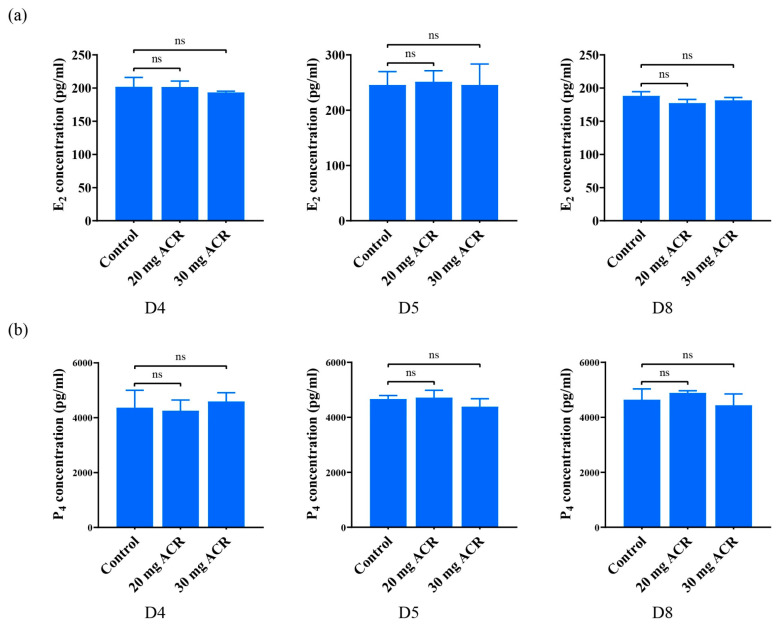
The concentrations of uterine E_2_ and P_4_ on days 4, 5, and 8 of pregnancy in mice treated with 0, 20 mg ACR/kg/d (20 mg ACR) and 30 mg ACR/kg/d (30 mg ACR) for 3 months, respectively. (**a**) The concentrations of uterine E_2_ on days 4, 5 and 8 of pregnancy. (**b**) The concentrations of uterine P_4_ on days 4, 5, and 8 of pregnancy. ns: no significant.

The coordination of estrogen and progesterone is essential for embryo implantation and decidualization [27]. Compared with the control group, the concentrations of E_2_ and P_4_ in the uterus of the ACR-treated groups did not change significantly on days 4, 5, and 8 of pregnancy (Figure 2a,b). The results suggested that ACR at least had no obvious effect on uterine E_2_ and P_4_ levels.

### 2.3. Acrylamide Treatments Affected Uterine Receptivity

**Figure 3:**
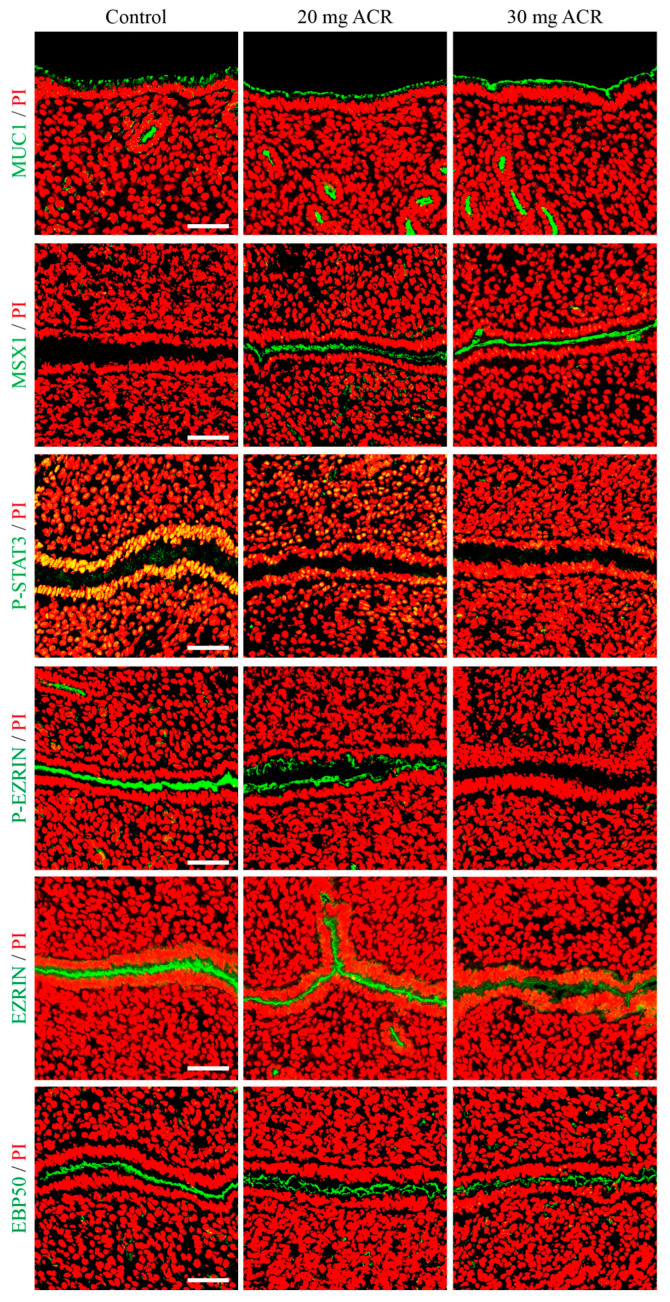
The immunofluorescence of MUC1, MSX1, p-STAT3, p-EZRIN, EZRIN, and EBP50 in mouse uteri on day 4 of pregnancy in mice treated with 0, 20 mg ACR/kg/d (20 mg ACR) and 30 mg ACR/kg/d (30 mg ACR) for 3 months. Green, the fluorescence signal of each molecule. PI, propidium iodide, for counterstaining nuclei in red fluorescence. Scale bar, 50 μm.

The downregulation of MUC1 and MSX1 is a marker of endometrial receptivity [28,29]. Compared to the control group, the levels of MUC1 and MSX1 immunofluorescence in luminal epithelium on day 4 of pregnancy were obviously increased in ACR-treated groups (Figure 3). The upregulation of p-STAT3, p-EZRIN, EZRIN, and EBP50 is also a marker of uterine receptivity [30,31,32]. On day 4 of pregnancy, the immunofluorescence of p-STAT3, p-EZRIN, EZRIN, and EBP50 was strongly detected in luminal epithelium in the control group, obviously reduced in the 20 mg/kg/d ACR-treated group, but barely seen in the 30 mg/kg/d ACR-treated group (Figure 3). The results suggested that ACR may compromise endometrial receptivity.

### 2.4. Acrylamide Suppressed Decidualization-Related Molecules

**Figure 4:**
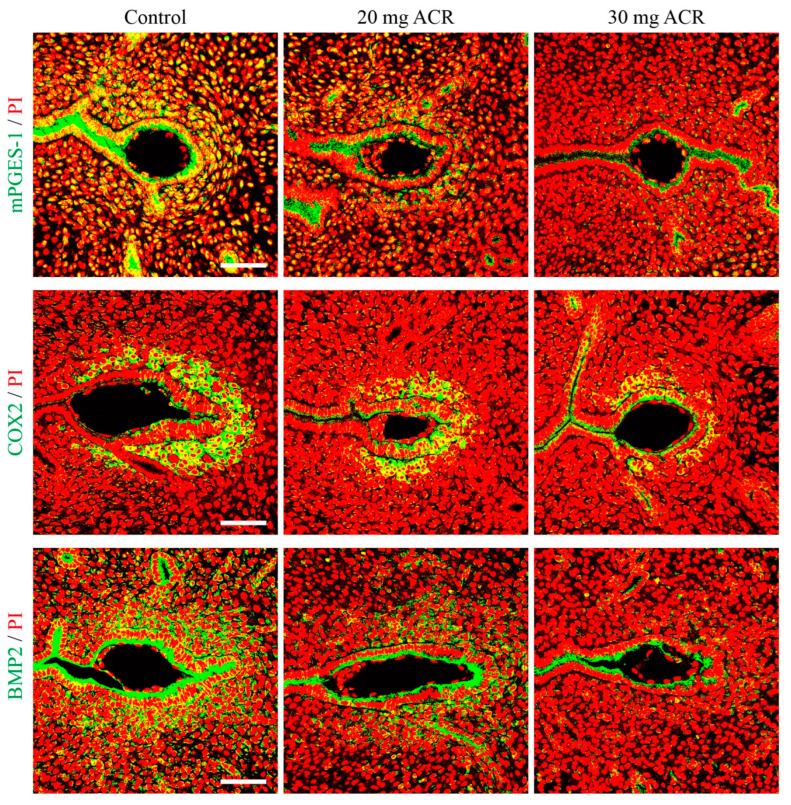
The immunofluorescence of mPGES-1, COX2, and BMP2 at implantation sites on day 5 of pregnancy in mice treated with 0, 20 mg ACR/kg/d (20 mg ACR), and 30 mg ACR/kg/d (30 mg ACR) for 3 months. Green, the fluorescence signal of each molecule. PI, propidium iodide, for counterstaining nuclei in red fluorescence. Scale bar, 100 μm.

mPGES-1, COX2, and BMP2 are key factors regulating decidualization and are strongly expressed in decidual cells at implantation sites [33,34,35,36]. On day 5 of pregnancy, the immunofluorescence of COX2, BMP2, and mPGES1 was strongly observed in the luminal epithelium and decidual cells surrounding the implanting blastocyst in the control group, moderately in the 20 mg/kg/d ACR-treated group, and weakly in the 30 mg/kg/d ACR-treated group (Figure 4). These results showed that acrylamide led to abnormal decidualization.

### 2.5. Acrylamide Inhibited Decidualization

**Figure 5:**
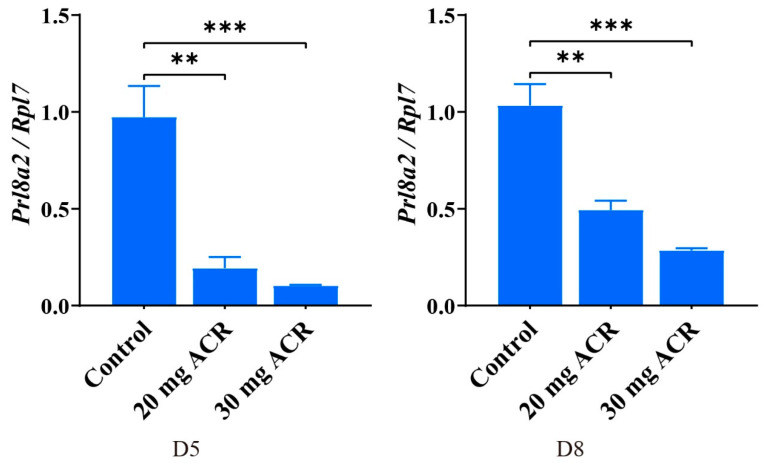
Uterine *Prl8a2* mRNA levels at implantation sites on days 5 and 8 of pregnancy in mice treated with 0, 20 mg ACR/kg/d (20 mg ACR), and 30 mg ACR/kg/d (30 mg ACR) for 3 months. ** *p* < 0.01; *** *p* < 0.001.

In order to further verify the effect of ACR on decidualization, we examined the *Prl8a2* levels of implantation sites on days 5 and 8 of pregnancy. The mRNA level of *Prl8a2*, a marker of mouse decidualization [37], was significantly reduced at implantation sites on days 5 and 8 by ACR treatment compared with the control group (Figure 5).

### 2.6. Acrylamide Caused Abnormal Expression of Oxidative Stress Molecules at Implantation Sites

**Figure 6:**
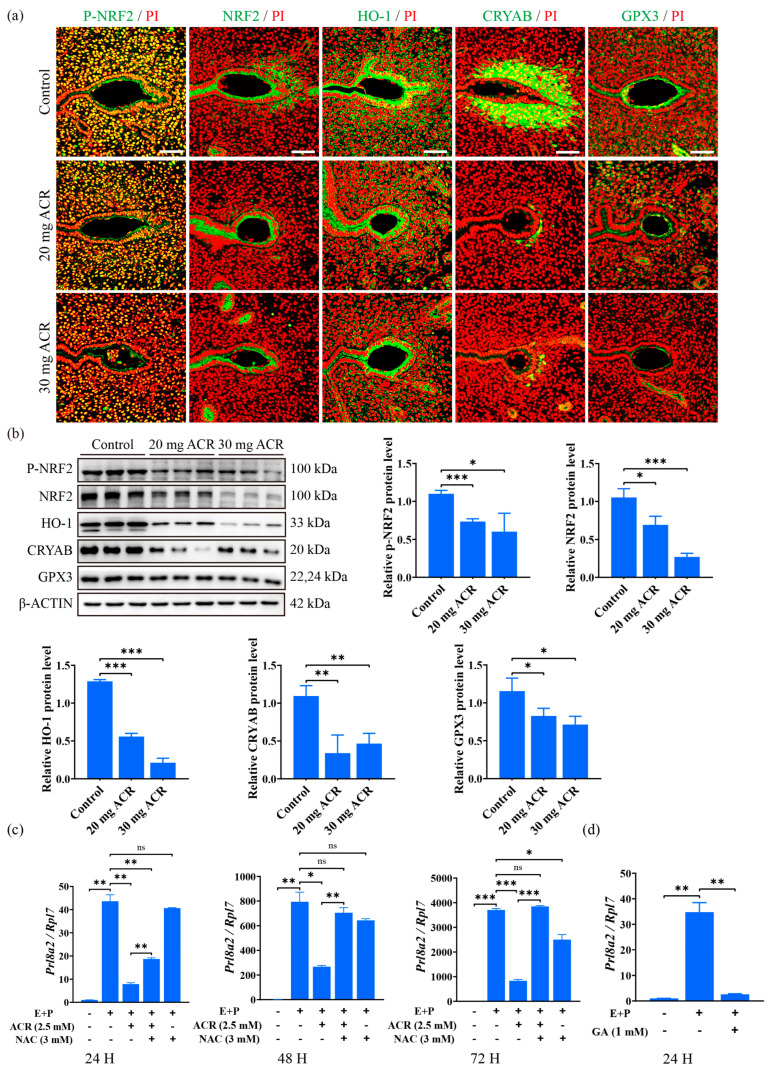
Effects of ACR on oxidative stress. (**a**) Immunofluorescence of p-NRF2, NRF2, HO-1, CRYAB, and GPX3 at implantation sites on day 5 of pregnancy in mice treated with 0, 20 mg ACR/kg/d (20 mg ACR), and 30 mg ACR/kg/d (30 mg ACR) for 3 months. Green, the fluorescence signal of each molecule. PI, propidium iodide, for counterstaining nuclei in red fluorescence. Scale bar, 100 μm. (**b**) Western blot analysis and quantification of p-NRF2, NRF2, HO-1, CRYAB, and GPX3 protein levels at implantation sites on day 5 of pregnancy in mice treated with 0, 20 mg ACR/kg/d (20 mg ACR), and 30 mg ACR/kg/d (30 mg ACR) for 3 months. (**c**) The *Prl8a2* mRNA levels in 2.5 mM ACR-treated mouse endometrial stromal cells were rescued by N-Acetylcysteine (NAC) under in vitro decidualization. (**d**) The *Prl8a2* mRNA level after mouse endometrial stromal cells under in vitro decidualization were treated with 1mM glycidamide (GA) for 24 h. * *p* < 0.05; ** *p* < 0.01; *** *p* < 0.001; ns: no significant.

p-NRF2, NRF2, HO-1, CRYAB, and GPX3 are important antioxidant molecules during oxidative stress, which are strongly expressed at the implantation site [38,39,40,41,42]. Compared with the control group, the immunofluorescence of p-NRF2, NRF2, HO-1, CRYAB, and GPX3 at the implantation site on day 5 of pregnancy was clearly downregulated (Figure 6a). Western blot analysis also revealed that the protein levels of p-NRF2, NRF2, HO-1, CRYAB, and GPX3 at implantation sites on day 5 of pregnancy were significantly decreased in ACR-treated groups (Figure 6b). N-Acetylcysteine (NAC) is a ROS inhibitor [43]. When mouse endometrial stromal cells under in vitro decidualization were treated with ACR for 24 h, 48 h, or 72 h, *Prl8a2* mRNA levels were significantly decreased, which were partially rescued by N-Acetylcysteine treatment (Figure 6c). Since ACR is metabolized to glycidamide (GA) [44], the mouse endometrial stromal cells were treated with GA for 24 h under in vitro decidualization. The *Prl8a2* mRNA level was significantly decreased by the GA treatment (Figure 6d). The results showed that acrylamide disturbed the expression of oxidative stress-related molecules during decidualization.

### 2.7. Acrylamide Caused Abnormal Expression of Ferroptosis-Related Proteins at Implantation Sites

**Figure 7:**
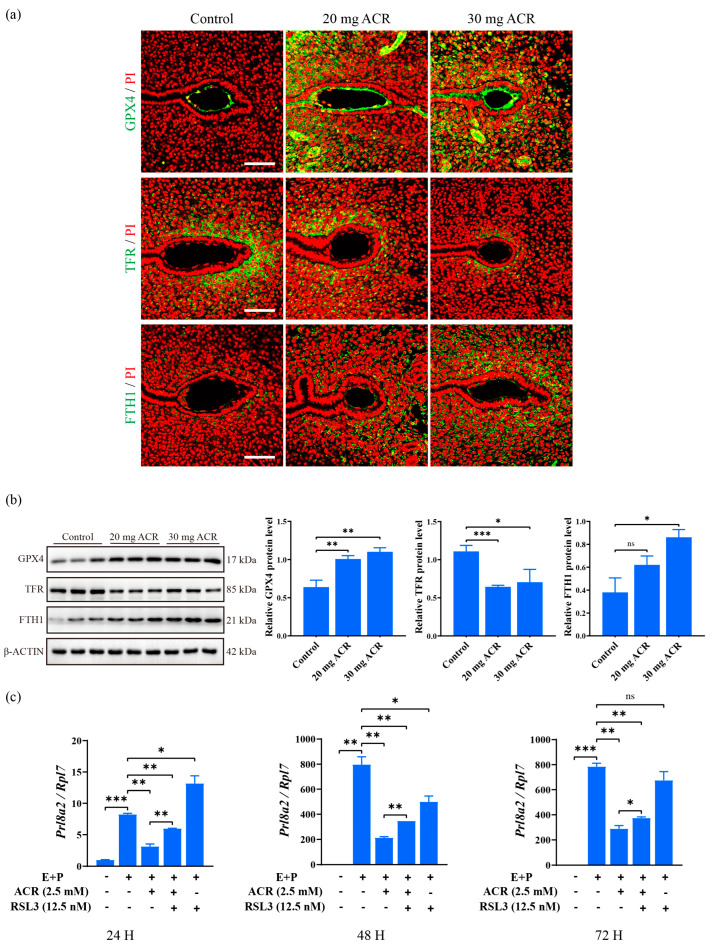
Effects of ACR on ferroptosis. (**a**) The immunofluorescence of GPX4, TFR, and FTH1 at the implantation site on day 5 of pregnancy in mice treated with 0, 20 mg ACR/kg/d (20 mg ACR), and 30 mg ACR/kg/d (30 mg ACR) for 3 months. Green, the fluorescence signal of each molecule. PI, propidium iodide, for counterstaining nuclei in red fluorescence. Scale bar, 100 μm. (**b**) Western blot analysis and quantification of GPX4, TFR, and FTH1 protein levels at the implantation site on day 5 of pregnancy in mice treated with 0, 20 mg ACR/kg/d (20 mg ACR), and 30 mg ACR/kg/d (30 mg ACR) for 3 months. (**c**) The *Prl8a2* mRNA levels in ACR-treated mouse endometrial cells were partially rescued by RSL3 treatment under in vitro decidualization. * *p* < 0.05; ** *p* < 0.01; *** *p* < 0.001; ns: no significant.

GPX4, TFR, and FTH1 are key players during ferroptosis [45,46]. Compared to the control group, GPX4 and FTH1 immunofluorescence at the implantation site was obviously increased in ACR-treated groups, while TFR immunofluorescence was decreased significantly (Figure 7a). GPX4 and FTH1 protein levels were also significantly upregulated at the implantation site on day 5 of pregnancy in ACR-treated groups, and TFR level was significantly downregulated (Figure 7b), suggesting that ferroptosis is suppressed by ACR treatment. RSL3 is a ferroptosis inducer [47]. *Prl8a2* levels under in vitro decidualization were significantly decreased by ACR treatment, which was partially rescued by RSL3 treatment (Figure 7c). These results showed that ACR disrupted the expression of ferroptosis-related molecules during decidualization.

## 3. Discussion

In this study, we showed that exposure of female mice to acrylamide led to a decrease in the number of implantations and litter size, suggesting that ACR was not conducive to pregnancy in mice.

ACR can be metabolized to glycidamide (GA) by GST and CYP2E1 enzymes in the mammalian body [44]. At least 6% of the ACR intake is converted to GA, which interacts with ACR to form a stable DNA adduct that can cause genotoxicity and even cancer [48]. The ACR molecule is small and hydrophilic, and is capable of passively diffusing throughout the body [49]. ACR is an electrophilic reagent that exerts its toxic effects by interacting with nucleophilic residues of macromolecules, especially cysteine residues [50]. Therefore, all tissues could theoretically be targets of ACR-induced toxicity. During pregnancy, the implantation of the embryo necessitates the transformation of the uterus into a receptive state. Failure to achieve this state may impede blastocyst attachment and result in compromised pregnancy outcomes [51]. In mice, ovarian estrogen and progesterone synergistically regulate endometrial receptivity [52]. ACR can affect the release of steroid hormones in the ovary by gavage, thereby significantly reducing the concentration of serum estrogen and progesterone [12]. However, in our study, ACR at least had no obvious effects on uterine estrogen and progesterone levels because the uterine concentrations of estrogen and progesterone exhibited no significant alterations following ACR treatment.

MUC1, a transmembrane glycoprotein, is downregulated at the apical surface of uterine luminal epithelium during embryo implantation and participates in the remodeling of endometrial function [53,54]. MSX1 is also decreased in uterine luminal epithelium during embryo implantation [28,51,55]. The upregulation of MUC1 and MSX1 in the endometrial epithelium on day 4 of pregnancy was not conducive to blastocyst attachment. Our results showed that the levels of MUC1 and MSX1 were upregulated in ACR-treated groups, which may indicate that ACR has a harmful effect on the implantation process. STAT3 phosphorylation at tyrosine 705 regulates epithelial cell proliferation and differentiation to maintain the biological conditions necessary for embryo implantation [30,56]. In our results, the level of p-STAT3 was downregulated in the uterine epithelium on day 4 of pregnancy after ACR treatment. p-EZRIN, EZRIN, and EBP50 also showed the same trend as p-STAT3, further indicating that ACR treatment compromises endometrial receptivity. These molecules are equally important in regulating endometrial receptivity. EZRIN is involved in cytoskeletal organization and intercellular adhesion in the endometrial epithelium [57]. Knockdown of EZRIN expression significantly inhibited trophoblast cell invasion, suggesting that EZRIN may promote trophoblast cell invasion during embryo attachment [58]. In addition, the absence of EBP50 severely disrupts the recruitment and distribution of EZRIN to the apical membrane, leading to villus formation and tissue defects, as EZRIN must bind to EBP50 to function [32,59]. The abnormal expression pattern of ERZIN and EBP50 in the ACR-treated groups is not suitable for embryo implantation.

The weight of the embryo implantation site can reflect the quality of decidualization to a certain extent [60]. A previous study reported that ACR significantly reduces the weight of the implantation site on day 8 and reduces the weight of artificially induced deciduoma in mice [13], suggesting that ACR exerts an influence on decidualization. In our study, ACR exposure resulted in a decrease in the weight of implantation sites on day 8 of pregnancy, and the *Prl8a2* mRNA level was significantly decreased at implantation sites on days 5 and 8 of pregnancy. Under in vitro decidualization, the *Prl8a2* mRNA level is also significantly inhibited by ACR treatments. These data further confirmed the detrimental effect of ACR on decidualization. In our study, the levels of mPGES-1, COX2, and BMP2 were decreased at implantation sites on day 5 of pregnancy by ACR treatment. mPGES-1 and COX2 play an important regulatory role in the decidualization process through synthesizing prostaglandin E2 [35,61]. Selective inhibition of COX2 leads to failure of decidualization along with reduced mPGES-1 expression [62]. As one of the upstream molecules of COX2, BMP2 is also critical for decidualization. Uterus-specific knockdown of BMP2 results in loss of decidual response and blockage of further embryo development in mice [34]. Therefore, the downregulation of these molecules also suggests that ACR can impair decidualization.

We examined the changes in oxidative stress and ferroptosis-related molecules because ACR can cause oxidative stress, and oxidative stress can further induce ferroptosis [22,63]. Our data suggested that the levels of p-NRF2, NRF2, HO-1, CRYAB, and GPX3 were decreased in the implantation sites on day 5 in ACR-treated mice. Ferroptosis-related molecules GPX4 and FTH1 showed an upregulated trend, while the TFR level was downregulated. Physiological levels of ROS play an important role during pregnancy [64]. Nrf2 is an antioxidant factor in the process of oxidative stress and protects tissues from ROS damage [65]. The reduction in NRF2 levels may result in increased inflammation and tissue damage. Due to the diminished antioxidant capacity, cells become susceptible to oxidative stress-induced damage [66,67]. Nrf2 gene ablation can aggravate the neurotoxicity of ACR in mice [68]. Our results indicated that the levels of p-NRF2 and NRF2 at implantation sites were downregulated, suggesting that ACR treatment enhanced oxidative stress during mouse decidualization. HO-1 is one of the key antioxidant genes regulated by NRF2 that can scavenge excess ROS [69,70]. In rats, ACR reduced HO-1 expression to increase oxidative stress [71]. Therefore, the decrease of HO-1 at implantation sites on day 5 may also indicate an increase in oxidative stress during mouse decidualization. CRYAB and GPX3 play an important antioxidant role during mouse decidualization [40,72]. The decreased expression of these antioxidant molecules may indicate that ACR increased oxidative damage during decidualization in mice. In our study, ACR-induced oxidative effects on in vitro decidualization could be partially rescued by NAC. This provided further evidence for the link between increased oxidative stress and impaired decidualization caused by ACR in mice.

GPX4 and FTH1 proteins are significantly decreased after ACR treatment [25]. GPX4 is an antioxidant enzyme that inhibits ferroptosis. When GPX4 activity is inhibited, lipid peroxidation accumulates, resulting in ferroptosis [73]. Through its iron oxidase activity, FTH1 converts toxic free Fe^2+^ into Fe^3+^ and stores it in the ferritin shell to prevent iron-mediated ROS production, maintain cellular iron homeostasis, and protect cells from oxidative damage [74,75]. However, our results demonstrate that the levels of GPX4 and FTH1 at the implantation sites of the ACR treatment groups were upregulated, suggesting that ferroptosis during embryo implantation and decidualization was suppressed by ACR treatment. The primary physiological function of TFR is to bind to transferrin, mediate the cellular uptake of iron, regulate the balance of intracellular iron levels, and maintain the body’s iron homeostasis [76,77]. The knockout of TFR in mice resulted in the loss of both embryos and placenta, as well as increased embryonic mortality [78]. Our results suggested that ACR treatment decreased the level of TFR, which may also suggest that ACR inhibited the ferroptosis process during mouse decidualization. In our study, RSL3, a ferroptosis inducer, was able to partially rescue ACR-induced damage on in vitro decidualization, suggesting a link between ferroptosis and decidualization.

In conclusion, our findings indicated that prolonged exposure to acrylamide led to dysregulation of oxidative stress- and ferroptosis-associated molecules during mouse implantation and decidualization.

## 4. Materials and Methods

### 4.1. Animals and Treatments

Mature ICR mice (6–8 weeks old) were purchased from Hunan Slaike Jingda Experimental Animal Co., Ltd. (Changsha, China), and kept in a temperature-controlled environment with a light cycle of 12 h. All animal protocols were approved by the Animal Care and Use Committee of Guizhou University (EAE-GZU-2023-T005). Female mice used in the experiment weighed 25 ± 1 g and were randomly divided into three groups: control group, 20 mg/kg/d group, and 30 mg/kg/d group. The body weight of female mice was measured weekly. The control group was provided with normal drinking water. The intake dose of ACR was calculated according to the body weight of the mice. Water consumption per cage was measured during the test to estimate the amount of ACR per kilogram of body weight in each mouse [79]. After all female mice were treated with 0, 20 mg/kg/d, and 30 mg/kg/d ACR for 3 months, female mice of each group were mated with fertile male mice aged 8–12 weeks to induce pregnancy (the day when vaginal plug was detected was defined as day 1 of pregnancy). The implantation site was determined on day 5 by intravenous injection of 0.2 mL of 1% Chicago blue dye (Sigma-Aldrich, St. Louis, MO, USA) dissolved in normal saline.

### 4.2. Immunofluorescence

Immunofluorescence was performed as previously described [80]. The uterine tissue was fixed with 4% neutral buffered formalin, dehydrated by alcohol gradient, and embedded in paraffin. Paraffin sections (5 μm) were deparaffinized, rehydrated, and the antigen was retrieved in citric acid or an EDTA solution. Sections were blocked with a 10% horse serum and incubated with the corresponding primary antibody at 4 °C overnight. Primary antibodies used in this study included MSX1 (1:200, BS-8512R, Bioss, Beijing, China), MUC1 (1:200, ab45167, Abcam, Cambridge, UK), p-STAT3 (1:200, ab76315, Abcam, Cambridge, UK), EZRIN (1:200, Cell Signaling Technology, Danvers, MA, USA), p-EZRIN (1:200, PA5-37763, Invitrogen, Carlsbad, CA, USA), EBP50 (1:200, 29771-1-AP, Proteintech, Wuhan, China), COX2 (1:100, 12282 T, Cell Signaling Technology), BMP2 (1:200, A0231, Abclonal, Wuhan, China), mPGES-1 (1:200, 160140, Cayman Chemical Company, Ann Arbor, MI, USA), CRYAB (1:200, ab281561, Abcam), HO-1 (1:200, 10701-1-AP, Proteintech), NRF2 (1:200, 80593-1-RR, Proteintech), p-NRF2 (1:200, DF7519, Affinity, Changzhou, China), GPX3 (1:200, ab256470, Abcam), GPX4 (1:100, ab125066, Abcam), TFR (1:200, ab214039, Abcam), and FTH1 (1:200, GTX101733, GeneTex, Irvine, CA, USA). After three washes with PBS, the sections were incubated with matched secondary antibodies (2.5 μg/mL, G21234, Invitrogen, Carlsbad, CA, USA) for 30 min at 37 °C, counterstained with propidium iodide (5 g/mL, PI, P4170, Sigma-Aldrich), and mounted with ProLong Diamond Antifade Mountant (Thermo Fisher’s, Waltham, MA, USA). Laser scanning confocal microscopy (Nikon, Tokyo, Japan) was used to collect the images.

### 4.3. Western Blot

Western blot was performed as previously described [81]. Tissues or cultured cells were lysed on ice in lysis buffer (50 mm Tris-HCl, pH 7.5; 150 mM NaCl; 0.25% sodium deoxycholate and 1% Triton X-100). The protein concentrations were quantified by the BCA method (Thermo Fisher Scientific, Waltham, MA, USA). The protein samples were separated on 10% or 12% SDS-polyacrylamide gel electrophoresis (SDS/PAGE) and transferred onto polyvinylidene difluoride (PVDF) membranes (IPVH00010, Millipore, Billerica, MA, USA). The membranes were blocked with 5% non-fat milk (BBI Life Sciences, Shanghai, China) for 1 h, and incubated with each primary antibody overnight at 4 °C and a matched secondary antibody. The signal was detected using the ECL chemiluminescence kit (Millipore, Saint Louis, MO, USA). Primary antibodies used in this study included CRYAB (1:1000, ab281561, Abcam), HO-1 (1:1000, 10701-1-AP, Proteintech), NRF2 (1:1000, 80593-1-RR, Proteintech), p-NRF2 (1:1000, DF7519, Affinity), GPX3 (1:1000, ab256470, Abcam), GPX4 (1:1000, ab125066, Abcam), TFR(1:1000, ab214039, Abcam), FTH1 (1:1000, GTX101733, GeneTex), and β-ACTIN(1:1000, 4967s, Cell Signaling Technology).

### 4.4. Isolation and Treatment of Mouse Endometrial Stromal Cells

Mouse endometrial stromal cells were isolated as previously described [82]. Mouse uteri on day 4 of pseudopregnancy were dissected longitudinally, washed with Hanks’ balanced salt solution (HBSS, H4891, Sigma–Aldrich), and digested with 6 mg/mL dispase II and 1% trypsin in HBSS at 4 °C for 1.5 h, at room temperature for 30 min, and at 37 °C for 10 min. The remaining uteri tissues were incubated with 0.15 mg/mL collagenase I (17100-017, Invitrogen, Houston, TX, USA) in HBSS at 37 °C for 35 min. Endometrial stromal cells were collected, plated in culture plates, and cultured with DMEM/F12 containing 10% FBS (040011A, Biological Industries, Cromwell, CT, USA). These cells were cultured in DMEM/F12 with 2% charcoal-treated FBS (cFBS, Biological Industries, Cromwell, CT, USA) 6 h before each treatment. The stromal cells were treated with estradiol-17 β (10 nM) and progesterone (1 µM) to induce in vitro decidualization. Estradiol-17 β and progesterone were prepared in anhydrous ethanol with the concentrations of 1 μM and 100 μM, respectively, and added into the culture medium at 1:100 (*v*/*v*). The same amount of ethanol was added to the control group. To analyze whether oxidative stress and ferroptosis are associated with impaired decidualization, the stromal cells treated with 2.5 mM ACR were rescued by 3 mM NAC (HY-B0215, MCE, Monmouth Junction, NJ, USA) or 12.5 nM RLS3 (HY-100218A, MCE) under in vitro decidualization. ACR and NAC were prepared in DMEM/F12 with the concentrations of 250 mM and 300 mM, respectively, and added into the culture medium at 1:100 (*v*/*v*). Glycidemide (GA, HY-119329, MCE), a metabolite of ACR, was used to treat stromal cells with 1 mM under in vitro decidualization. RSL3 and GA were prepared in DMSO with the concentrations of 2.5 μM and 200 mM, respectively, and were added to the culture medium at 1:200 (*v*/*v*). For each control group, the same amount of each solvent was added to the culture medium.

### 4.5. Real Time RT-PCR

qPCR was performed as previously described [83]. The total RNAs were extracted using the Trizol Reagent Kit (9109, Takara, Kusatsu, Japan), digested with RQ1 deoxyribonuclease I (Promega, Fitchburg, WI, USA), and reverse-transcribed into cDNA with the Prime Script Reverse Transcriptase Reagent Kit (Takara, Japan). For real-time PCR, the cDNA was amplified using a SYBR Premix Ex Taq Kit (Q311-02-AA, Vazyme, Nanjing, China) on the CFX96 Touch Realtime System (Bio-Rad, Irvine, CA, USA). Data were analyzed using the 2^−ΔΔCt^ method and normalized to the mouse Rpl7 level. The primers were designed and synthesized by Shanghai Sheng gong Bioengineering Co., Ltd (Shanghai, China). The primer sequences used in this study are as follows: *Prl8a2* (NM_010088, mouse): 5′-AACAGGAGAGAATGGCTGCTC-3′ and 3′-AACAGGAGAGAATGGCTGCTC-5′. *Rpl7* (NM_29016, mouse): 5′-GCAGATGTACCGCACTGAGATTC-3 and 3′-ACCTTTGGGCTTACTCCATTGATA-5′. Every experiment was carried out at least three times.

### 4.6. Measurement of E_2_ and P_4_ Concentration

The uterine tissue was homogenized and the supernatant was collected by centrifugation. Protein concentration was measured to standardize each sample. Mouse E2 (CSB-E07280m, Cusabio, Wuhan, China) and P4 (E-OSEL-M0006, Elabscience, Wuhan, China) ELISA kits were used to analyze the concentration of E2 and P4 in uterine supernatant. Experimental procedures were executed in accordance with the assay kits manufacturer’s protocol.

### 4.7. Statistical Analysis

The data were analyzed using GraphPad Prism 9.0 software. Student’s *t* test was used to examine the differences between the two groups. One- or two-way analysis of variance (ANOVA) test was used to compare multiple groups. Quantitative analysis of protein bands was performed using ImageJ software (Win 64). There were at least three mice per group. The data were presented as mean ± standard deviation (SD). Statistical significance was defined as *: *p* < 0.05; **: *p* < 0.01; ***: *p* < 0.001, ns: not significant.

## Data Availability

All data needed to evaluate the conclusions in the paper are presented in the paper. All materials in this manuscript are available from the corresponding author on reasonable request.
